# A New Silicon Mold Process for Polydimethylsiloxane Microchannels

**DOI:** 10.3390/mi15070848

**Published:** 2024-06-29

**Authors:** Lung-Jieh Yang, Sameer Shaik, Neethish Kumar Unnam, Valliammai Muthuraman

**Affiliations:** 1 Department of Mechanical and Electromechanical Engineering, Tamkang University, New Taipei 251301, Taiwan; shaiksameer.munna840@gmail.com (S.S.); vamsi.neethish@gmail.com (N.K.U.); 2 Department of Electronics and Communication Engineering, Vel Tech Rangarajan Dr Sagunthala R&D Institute of Science and Technology, Chennai 600062, India; drvalliammaim@veltech.edu.in

**Keywords:** PDMS microchannel, laser cutting, anodic bonding

## Abstract

As an alternative to SU-8 soft lithography, a new silicon mold process of fabricating PDMS microchannel chips was proposed. A picosecond laser is used to cut through a 550 μm thick silicon wafer and generate the original microchannel pattern with a 50 μm minimum feature size. This single-crystal silicon pattern, with the edge debris caused by laser cutting being trimmed off by a KOH solution and with the protection field oxide layer being removed by BOE afterwards, firmly resided on a glass substrate through the anodic bonding technique. Four-inch wafers with microchannel patterns as the PDMS mold cores were successfully bonded on Pyrex 7740 or Eagle XG glass substrates for the follow-up PDMS molding/demolding process. This new maskless process does not need a photolithography facility, but the laser cutting service must be provided by professional off-campus companies. One PDMS microchannel chip for particle separation was shown as an example of what can be achieved when using this new process.

## 1. Introduction

Since the boom of MEMS in the late 1980s, many universities and research institutes have invested a lot of money to build clean rooms required for MEMS processes and purchased micromachining equipment. People have successively developed various sensors and actuator components, which are expected to be published, commercialized, and marketed. However, due to the increasing operation/maintenance costs and the fact that process knowledge is not easy to manage and pass on over a long time, many old MEMS facilities are facing retirement.

In order to continue the development of MEMS technology and new components without high-cost MEMS clean room facilities, the below considerations may be needed to simplify the manufacturing process.

First, people can look for foundry services for the MEMS process. The MEMS research team of a university, like an IC design house company, is only engaged in the design of MEMS components, and then they pay or entrust a professional complementary metal–oxide–semiconductor (CMOS) foundry line to complete MEMS component fabrication. Finally, they receive MEMS chips for testing, verification, performance improvement, and subsequent engineering applications [1,2,3]. For example, the Taiwan Semiconductor Research Institute (TSRI) provides the U18 CMOS MEMS process for free foundry in the academic world. The outer appearance of a MEMS structure is only a CMOS suspended membrane with a cavity depth of 45 μm, and the entire membrane area is filled with 8 μm × 8 μm etched holes. This MEMS configuration can be directly applied to the design of, e.g., flow sensors [4]. But to design other MEMS components, such as pressure sensors and inertial sensors, some specific post processes are required to implement the fabrication. Therefore, it is not easy to achieve the ideal of fabless foundry without an expensive clean room facility.

Second, researchers have been trying to develop a more maskless process [5,6]. Without a photomask, there is less need for lithography facilities, and the demand and budget for clean room facilities will be minimized. For example, the aforementioned CMOS MEMS foundry process produces pressure sensor chips for which a polyparaxylene (parylene) coating process needs to be added [7]. The 8 μm × 8 μm etched holes of the suspended membrane can be sealed to create a vacuum compartment with a depth of about 30~40 μm below the CMOS structure. Another example is the CMOS MEMS foundry process which can make suspended metal microstructures. The isotropic dry etching of fluoride-based plasma is a process that can be used to remove the inter-layer dielectrics (ILD) oxide among metal layers within the pre-specified etching window on the CMOS chip. This methodology to free-stand suspended metal microstructures is also a maskless process that does not need photolithography [8,9,10]. A prior study using image-guided in situ maskless lithography (IGIs-ML) was also reported recently [11].

**Figure 1:**
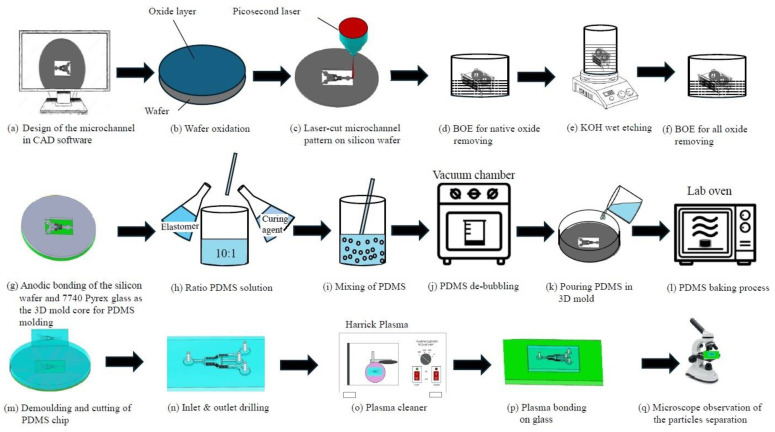
A new maskless process flow for PDMS microchannels.

Third, there are many microfluidic chips, and their manufacturing process applies to the field of BioMEMS or microfluidics [12,13]. The general polydimethylsiloxane (PDMS) microchannel process includes the fabrication of microfluidic mold cores, PDMS casting/demolding, and room temperature bonding with glass substrates. The last two parts, PDMS casting/demolding, are shown in Figure 1h–n, and they do not require an ultraclean laboratory environment; only a laminar flow hood, deionized (DI) water, a nitrogen gas gun, and some chemicals are needed. The plasma surface treatment machine required for PDMS bonding with the glass substrate at room temperature is shown in Figure 1o,p; it is a stand-alone vacuum chamber that can be operated in a general air-conditioned laboratory. Only as the microfluidic mold is made, it generally adopts the SU-8 thick photoresist process, and the clean room equipment used for photolithography is still indispensable. In addition, 3D-printed PDMS molds [14], which are good for screw-like or spiral molds, provide another possibility for fabricating nonplanar PDMS microchannels.

To develop a new PDMS microchannel process rather than using a photolithography facility and 3D printing, the authors’ research group published a process proposal in 2022 combining laser cutting and anodic bonding [15]. A monocrystalline silicon mold replaces SU-8 photoresist and its lithography process, as shown in the three steps in Figure 1a–c,g. Figure 1c shows how laser processing is used to cut the same microchannel profile as the original SU-8 photoresist on a silicon wafer. By removing the unwanted part on the silicon wafer, the silicon microchannel patterns were anodic bonded to a glass substrate, as shown in Figure 1g, which is used as a strong mold core for subsequent PDMS molding, as shown in Figure 1h–n. This silicon-on-glass mold outperforms SU-8 photoresist in terms of repeated molding times, cleaning durability, mechanical collision resistance, and chemical corrosion resistance.

The laser cutting process shown in Figure 1c is also a foundry service using a picosecond laser or even a femtosecond laser commissioned by a professional off-campus company, and the minimum feature size of processing is about 50 μm. Although the spatial resolution does not reach the level of 1~10 μm, it can be processed through the 550 μm thickness of 4-inch silicon wafers. Therefore, the finished microchannel aspect ratio increases to 11, which is not inferior to SU-8 micro patterns. As a result, the depth of the microfluidic channel has been increased to 550 μm, which is comparable to the maximum thickness provided by SU-8 photoresist [16]. The running cost of laser cutting foundry is not low but still comparable to the production cost of chrome photomasks. Anyhow, it can save the cost of operating a clean room, photolithography, and the need to purchase SU-8-related consumables. Preparing anodic bonding equipment, as shown in Figure 1g, is not particularly difficult and expensive, and this equipment can be purchased and assembled by a single person with a 1 kV DC high-voltage power supply, a 600 °C infrared (IR) hot plate, and a graphite conductive heating pad.

The results of the new PDMS microchannel process used in ref. [15] show that there is obvious debris residue on the edge of the cutting contour of the silicon wafer after the laser cutting process. The height of the edge debris exceeds the thickness of the silicon wafer and protrudes above the surface of the silicon wafer. The debris actually hinders the subsequent process of anodic bonding, making the bonding production yield and the bonding strength lower than those in the ideal case without edge debris. This paper specifically addresses this issue by adding a KOH wet etching procedure, shown in Figure 1d–f, in the hope of effectively removing residual silicon debris.

## 2. Materials, Methods, and Results

As shown in Figure 1e, this paper proposed using a KOH solution (85% in purity, Echo Chemical Co., Ltd., Miao-Li, Taiwan) [17,18] to remove the silicon debris of the laser cutting edges. The materials and parameters of the new maskless process described herein are outlined below.

- Laser cutting [19,20]

An innovative micromachining method that has transformed the production of electronic components and other microdevices is picosecond laser cutting on a silicon wafer. It makes precise use of ultrashort laser pulses, usually lasting picoseconds (10^−12^ s), to remove material from the silicon wafer. This technology is a useful tool in the semiconductor sector and other high-precision production industries because it has several benefits over conventional cutting processes. The picosecond laser cutting mechanism is based on nonlinear absorption processes in silicon. The quick creation of free electrons and the development of plasma result from multi-photon absorption and avalanche ionization caused by the strong picosecond laser pulse’s interaction with silicon. The remaining laser energy is absorbed by this plasma, which is made up of free electrons and ionized atoms. It then quickly expands, ejecting material from the surface. Because of the laser pulse’s brief length, its heat dispersion is reduced, and nearby areas are spared from harm. The energy is deposited into a highly confined zone. The picosecond laser machine used herein is ML-UV pico 580 (Micron Laser Co., Ltd., New Taipei City, Taiwan). Its wavelength is 355 nm, and its pulse laser power setting is 15 W. The laser ablation loses about 100 μm in width of silicon which should be compensated by shifting the cutting path 50 μm away from the original design path along both sides of the microchannel contour.

- KOH and BOE etching

Potassium hydroxide (KOH) wet etching is one of the popular techniques in silicon bulk micromachinings to etch single-crystalline silicon (SCS) in a selective manner. It makes use of the anisotropic properties of SCS etching in KOH, where the crystallographic orientation has a major impact on the etch rate variation. This makes it possible to create accurate, well-defined geometric constructions with predetermined proportions and forms. A protective masking layer—typically silicon dioxide or silicon nitride—is patterned onto the wafer’s surface. Depending on the orientation and pattern of the mask, the etch rate is the quickest in the crystallographic direction, resulting in the production of pyramidal pits or V-shaped grooves [21]. In this study, KOH etching is not used to make specific V-grooves but to trim out the edge debris caused by the previous picosecond laser cutting process. Because most of the SCS surface, except for the laser-cut edge debris, was protected with SiO_2_, KOH etching only occurred on the edge debris, and it is a maskless process. The temperature, etching duration, and KOH concentration are 60 °C, 19 min, and 33 weight %, respectively. The etching rate of SCS on the {100} surface was calculated as 0.63 μm/min, and the etched width of the SCS including the edge debris was measured as 12 μm [17,18]. 

**Figure 2:**
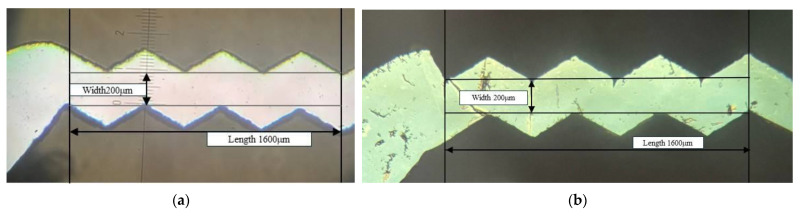
The laser-cut microchannel patterns and the edge debris removal process: (**a**) before KOH etching; (**b**) after KOH etching.

For precise control of the etching time and to minimize the etching non-uniformity or the undercut errors of KOH anisotropic etching, a 10 s buffered oxide etch (BOE) dipping process, shown in Figure 1d, for removing the native oxide of the debris surface before the KOH etching process is necessary to initiate the counting of the exact duration of the KOH etching process. (It generates many hydrogen bubbles as the silicon is subjected to the chemical reaction of KOH etching to denote the initiation. To avoid possible bad roughness and pyramid formation due to hydrogen bubble adhesion during KOH etching [22], it is better to substitute tetramethyl ammonium hydroxide (TMAH) for KOH [23].) Accordingly, to protect the most non-edge area of the silicon wafer surface from KOH etching, the purchased silicon wafers should be grown with a 0.3 μm thick field oxide in advance, and then, they should be cut by lasers, as shown in Figure 1c. After the KOH solution removes the edge debris, as shown in Figure 1e, the silicon sample is dipped in BOE again to strip the entire oxide layer, as shown in Figure 1f, so as to continue the anodic bonding process, as shown in Figure 1g. The etching rate of the field oxide in BOE under 25 °C is within the range of 0.08~0.12 μm/min [24], and the stripping time for removing 0.3 μm thick oxide should be within the range of 2.5~3.75 min. However, to avoid the chemical aging issue of BOE, a longer oxide-stripping time of 4 min should be used. The laser-cut microchannel patterns and the edge debris removal process are shown in Figure 2.

- Si-7740 glass anodic bonding [25]

**Figure 3:**
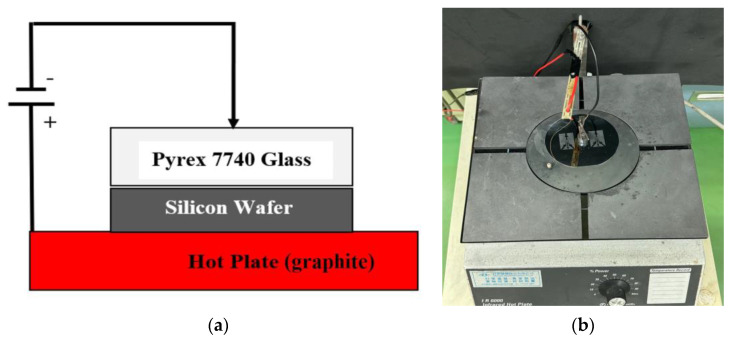
Self-made anodic bonder. (**a**) Setup schematic; (**b**) real picture of anodic bonder with bonded wafer.

Anodic bonding is a wafer bonding technique used to produce a hermetic seal between silicon and glass substrates. It is sometimes referred to as electrostatic bonding or field-assisted bonding. Because of its strong binding strength, excellent dependability, and compatibility with a variety of materials, it is a process that is frequently used in MEMS. Pyrex 7740 (Changsha Anole Precision Glass Co., Ltd., Hunan, China), a glass that is most frequently used in this method, is recognized for having electrical and thermal characteristics that make it perfect for anodic bonding with silicon. As shown in Figure 3, the glass side is the negative electrode, and the silicon side is the positive electrode of a wafer stack that is subjected to a high DC voltage, often set as 850 V. The assembly is heated to a temperature of 450 °C simultaneously (with a glass thickness of 500 μm). Generally speaking, anodic bonding takes a few minutes for 4-inch wafers. Recently, Corning Corp. (Corning, NY, USA) stopped the production of Pyrex 7740 glass. Eagle XG glass substrates can also undergo the anodic bonding process but need more strict bonding conditions [26].

**Figure 4:**
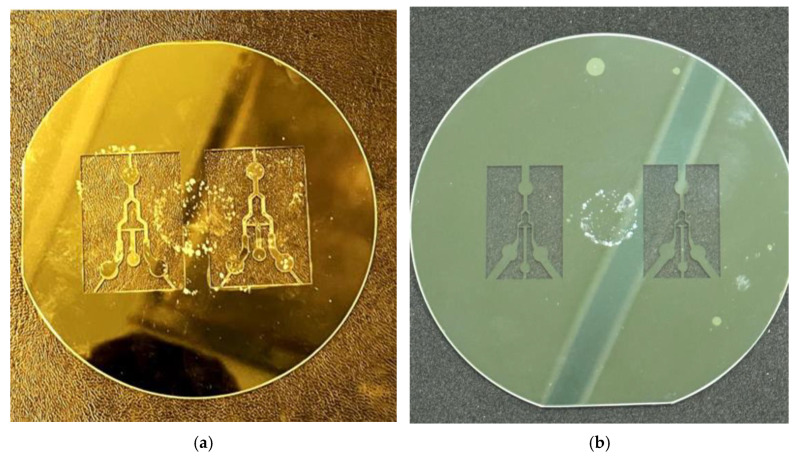
Microchannel mold after anodic bonding: (**a**) 4-inch silicon wafer with edge debris and consequent non-bonded area at light color microchannel [15]; (**b**) modified sample with uniform color in this work.

In general, anodic bonding can be carried out even when the bonding environment is not a high-quality clean room. However, if the laser-cut wafer is not flat enough even with the edge debris on the silicon wafer, as shown in Figure 4a, those local areas with the microchannel patterns cannot be successfully anodic-bonded with the glass substrate [15]. Figure 4b shows the modified sample without edge debris and the perfectly bonded surface in this work.

- PDMS molding/demolding

A flexible and popular technology for producing flexible electronics, microfluidic devices, and other complex structures is PDMS molding and demolding. The procedure entails employing PDMS, which is renowned for its optical clarity, biocompatibility, and simplicity of manufacture, to create a negative duplicate of a master mold. The first step in the molding process is to prepare a master mold, which is usually made on a silicon wafer using SU-8 photolithography or the laser cutting technology presented in this work. The pattern or structure intended to be duplicated in PDMS is present in the mold. To aid in subsequent demolding, a releasing agent—such as a silane compound—is placed on the mold surface. After mixing Sylgard 184 A and B in a precise ratio—typically 10:1—the PDMS mixture is degassed to eliminate air bubbles. After that, the mixture is put into the master mold and allowed to cure for a few hours at a temperature between 65 °C and 85 °C. The cross-linking that occurs during curing causes the PDMS prepolymer to solidify and take on the desired pattern that was copied from the master mold. The final PDMS structure’s mechanical characteristics may be controlled by varying the curing time and temperature. After curing, the PDMS duplicate must be carefully removed from the master mold during the demolding process. This procedure is facilitated by the releasing agent that was previously applied, which keeps the PDMS from sticking to the mold too firmly. The PDMS duplicate might need to undergo additional substrate bonding in the next section. PDMS molding’s adaptability and simplicity of usage make it a useful tool for microfluidics and BioMEMS. 

**Figure 5:**
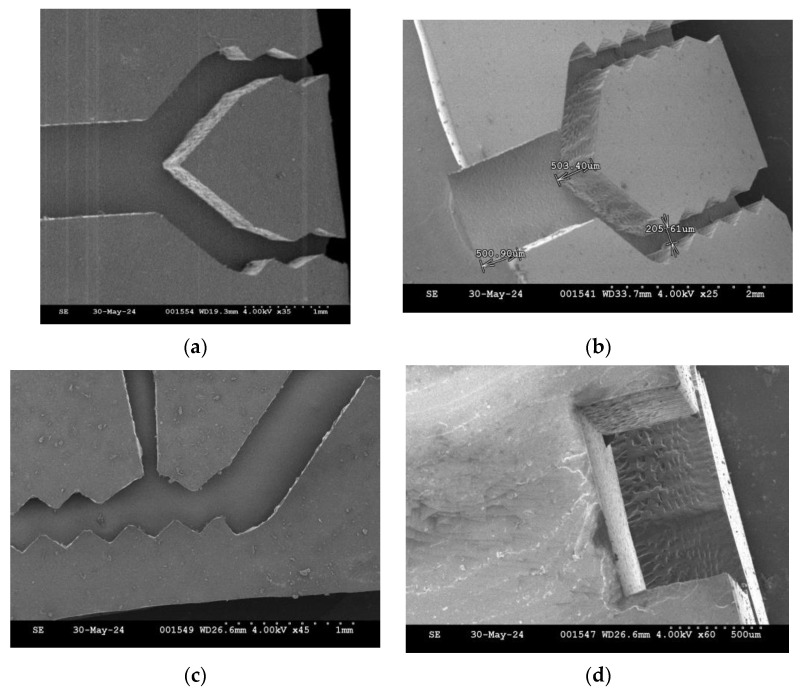
SEM photos of the fabricated PDMS microchannel chips with U-shaped grooves (**a**) with a depth of 300 μm at the entrance and (**b**) with a depth of 500 μm at the entrance. (**c**) The zigzag channel pattern; (**d**) the vertical side wall or the cross-section view of the U-shaped groove.

Figure 5a,b show SEM photos of the fabricated PDMS microchannel chips with 300 μm and 500 μm deep U-grooves, respectively, which were obtained using this new maskless process. Figure 5c shows that the geometry of the zigzag channel pattern is still preserved well without debris and not too much undercut on the channel edge. Figure 5d shows the acceptable surface morphology on the vertical side walls of the U-grooved channels.

- PDMS–glass bonding [27]

The most popular technique for attaching PDMS to glass is surface activation using an oxygen plasma treatment. During this procedure, oxygen plasma is applied to the surfaces of the PDMS and glass, causing silanol (Si–OH) groups to be introduced onto them. When the two surfaces come into contact, the highly reactive silanol groups easily form siloxane (Si–O–Si) linkages. In order to create a good bond, the plasma treatment parameters are essential. The PDC-001 HP plasma cleaner system (Harrick Plasma, NY, USA) was used. The radiofrequency (RF) power is 100 W. The pressure ranges from 200 mTorr to 1 Torr. The samples’ exposure time to achieve PDMS–glass bonding is 30 s herein. This surface treatment, which is performed before PDMS–glass bonding, could use both oxygen plasma and the plasma.

**Figure 6:**
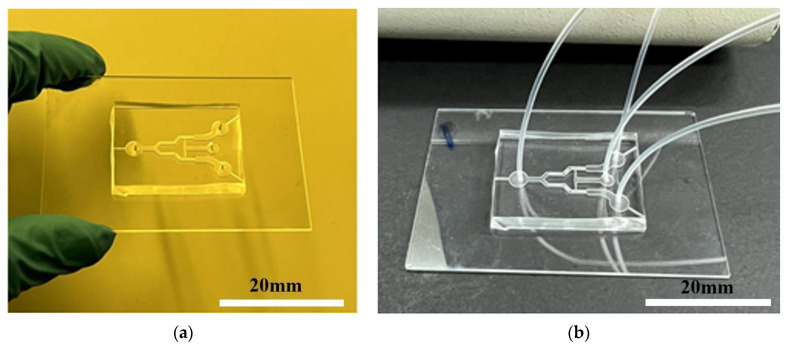
PDMS microchannel chips after PDMS–glass bonding using plasma treatment. (**a**) PDMS–glass chip sample; (**b**) chip sample connected with inlet and outlet piping.

The bonding process is started as soon as the PDMS and glass surfaces are aligned and pushed together after the plasma treatment. Strong and irreversible covalent bonds are formed between the surfaces as a result of the quick development of siloxane bonds. The bonded PDMS–glass assembly is usually subjected to a post-bonding annealing procedure to further strengthen the connection. This entails heating the assembly for a certain amount of time, usually between 60 and 100 °C. This heat treatment encourages the diffusion of PDMS chains onto the glass surface. Figure 6 shows the PDMS microchannel chips after the PDMS–glass bonding process.

**Figure 7:**
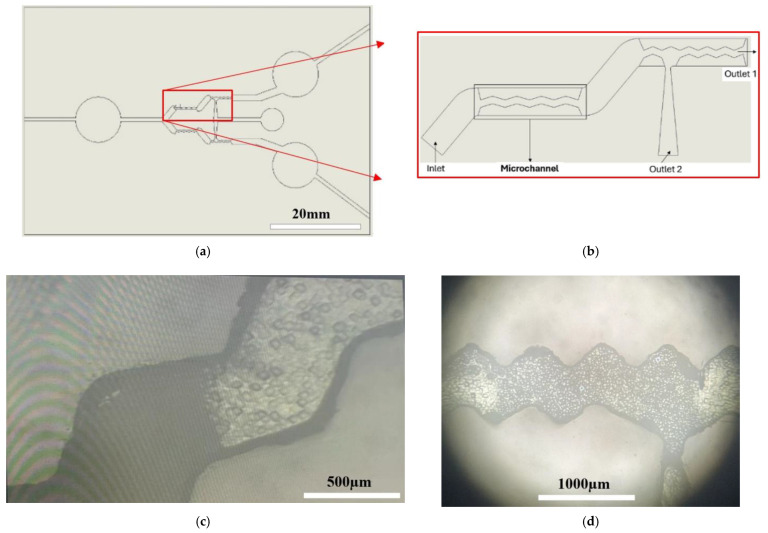
PDMS microchannel chips with depth of 500 μm when releasing particles for separation (using Olympus OLS4100 3D confocal microscope): (**a**) whole contour of PDMS chip including grooved microchannel and inlets; (**b**) magnified microchannel part of 6 mm in length; (**c**) particle aggregation at grooved area; (**d**) at Outlet 2.

Figure 7 shows the use of the new process in this work. This example of fabricated PDMS microchannel chips with a 500 μm channel depth can be used for particle separation. Different from the authors’ prior work in ref. [28], the simplified zigzag microchannels shown in Figure 7b replaced the previous cilia microchannels. They also allow the particle flow to move in a wavy manner under the flow speed of 0.5~2.0 m/s and separate particles of different sizes into two outlets. Most of the bigger beads move to Outlet 1; the smaller beads move to Outlet 2. This zigzag microchannel contour is easier to fabricate using the laser cutting method presented in this work, and the particle separation efficiency is still similar to the prior cilia design. An Olympus OLS4100 3D confocal laser microscope was utilized to carry out the non-contact 3D-like observation of the particle separation experiment. The filling solution needed for the test is mixed using 200 mL of water and 2 g of micro-silica dimer SiliaFlash^®^ C60 (SiliCycle Inc., Quebec City, QC, Canada), which have particles that range in size from 5 to 20 µm. A KdScientific KDS-100 syringe pump was used to drive the filling flow. Figure 7c,d depict particle aggregation at different places inside the microchannel after 3 min of the filling flow experiment. More detailed results regarding separation and discussion will be mentioned in the authors’ future papers.

## 3. Conclusions

A maskless process of fabricating PDMS microchannel chips was demonstrated by using a picosecond laser to cut 4-inch silicon wafers of 550 μm in depth to generate microchannel patterns. Through proper KOH solution trimming and being anodically bonded to glass substrates, this new method provides robust PDMS mold cores for microchannel chip fabrication without the need for a photolithography facility. By using fabricated U-grooved microchannels with less undercut and acceptable surface morphology on the vertical side wall, diverse PDMS microchannel chips can be developed using this new method.

## Data Availability

Dataset available on request from the authors.
